# *In Vivo* and *In Vitro* Antinociceptive Effect of *Fagopyrum cymosum* (Trev.) Meisn Extracts: A Possible Action by Recovering Intestinal Barrier Dysfunction

**DOI:** 10.1155/2012/983801

**Published:** 2012-12-17

**Authors:** Lina Liu, Xueting Cai, Jing Yan, Yi Luo, Ming Shao, Yin Lu, Zhiguang Sun, Peng Cao

**Affiliations:** ^1^Institute of First Clinical Medicine, Nanjing University of Chinese Medicine, Jiangsu, Nanjing 210046, China; ^2^Department of Liver Disease, The Affiliated Hospital of Nanjing University of Chinese Medicine, Jiangsu, Nanjing 210029, China; ^3^Laboratory of Cellular and Molecular Biology, Jiangsu Province Institute of Traditional Chinese Medicine, Jiangsu, Nanjing 210028, China; ^4^Institute of Pharmacy, Nanjing University of Chinese Medicine, Jiangsu, Nanjing 210046, China

## Abstract

*Fagopyrum cymosum* (Trev.) Meisn (Fag) is a herb rhizome which has been widely used to treat diseases. To investigate the effects and mechanisms of the Fag on irritable bowel syndrome (IBS), *in vivo* neonatal pups maternal separation (NMS) combined with intracolonic infusion of acetic acid (AA) was employed to establish IBS rat models. Fag reduced their visceral hyperalgesia and the whole gut permeability, ameliorated colonic mucosa inflammation and injury, and upregulated the expression of decreased tight junction proteins (TJs) of claudin-1, occludin, and ZO-1 (except ZO-2) in colonic epithelium. Caco-2 monolayer cells were incubated with TNF-**α** and IFN-**γ** *in vitro* to establish an epithelial barrier dysfunction model whose transepithelial electrical resistance (TER) depended more on dose of Fag than that of the controls, and whose TJs levels were lower than those of the controls. Fag upregulated the NP-40 insoluble and soluble components of the four TJs markedly in a dose-dependent manner. These data suggest that Fag alleviated the hyperalgesia of IBS rats by reducing intestinal inflammation and enhancing mucosal epithelial function after regulating the structure and function of TJs.

## 1. Introduction

Irritable bowel syndrome (IBS) is a chronic functional bowel disorder featured in abdominal pain and disturbed bowel habits. One of the pathomechanism is gastrointestinal motility dysfunction accompanied by visceral hyperalgesia [1]. Recent studies have found that chronic low-grade mucosal inflammation potentially led to IBS [2], as well as coexistent immune abnormalities. The T lymphocytes in descending colon mucosa significantly increased in the case of postinfectious IBS (PI-IBS) and non-PI-IBS [3]. Furthermore, the cytokine concentrations in the colonic mucosa of IBS patients increased due to activated immune system [4], and expression of mast cells adjacent to intestinal plexus in non-PI-IBS patients was highly expressed, which [2] resulted in hyperalgesia.

It has been reported that the intracolonic infusion of supernatants from the colonic biopsies of IBS patients led to somatic and visceral hyperalgesia [5] as well as impaired colonic permeability and sensitivity of mice [6]. Repeated stress increased CD4^+^/CD8T^+^ cells [7] and IFN-*γ*, which directly affected the tight junction proteins (TJs), and altered colonic mucosal barrier functions eventually [8]. However, adding supernatants of the culture from human colonic biopsies to Caco-2 cells reduced transepithelial resistance (TER), decreased ZO-1 mRNA in Caco-2 evidently, and increased paracellular permeability (PP) which correlated with abdominal pain [9]. Dunlop [10] and Spiller [11] have demonstrated that the intestinal permeability in PI-IBS and non-PI-IBS subgroups of diarrhea-predominant IBS elevated. Kong et al. [12] also showed that downregulated claudin-1 and -4 expressions were associated with changed defecation habits of D-IBS patients [13]. Finally, intestinal mucosal inflammation may contribute to altering TJs structures and PPs in IBS patients.

*Fagopyrum cymosum* (Trev.) Meisn (Fag), which is a herb rhizome of the Polygonaceae family and buckwheat species, has been widely used to treat bacterial dysentery, menorrhalgia and abdominal pain in Chinese medicine. Our previous clinical practices have revealed that Fag effectively mitigated abdominal pain, diarrhea, and bloating of IBS, and we have a Chinese authorized patent on Fag treatment of IBS. We assumed that Fag alleviates hyperalgesia of IBS by preventing intestinal mucosal immune disorders or decreasing colonic permeability via affecting TJs. Thereby motivated, we extracted the active ingredients of Fag and established animal and cell models to verify the hypothesis and to clarify the pharmacological targets and mechanisms.

## 2. Materials and Methods

### 2.1. Animal Experiments

#### 2.1.1. Preparation of Fag Extracts

Fag was provided by Nantong Jinghua Pharmaceutical Co., Ltd., Jiangsu Province of China, and was extracted by conventional refluxing in 3 fold of 50% ethanol for 3 h, 2 fold of 50% ethanol for 2 h, and 1.5 fold of 50% ethanol for 1 h, respectively. Then the product was decompressively concentrated and then spray-dried. The resulting powders were dissolved in 1% NaOH, the pH of which was then adjusted to 7.4 by HCl. The product was then diluted to a constant volume by saline and finally filtered prior to sterilization. The samples were identified by the Jiangsu Provincial Institute for pharmaceutical inspection of China.

#### 2.1.2. Animals and Neonatal IBS Modeling

Neonatal Sprague-Dawley rats weighing 5–6 g in average were provided by the Animal Center of Nanjing University of Chinese Medicine. Experiments were conducted in accordance with the standards of Animal Ethics Committee of Nanjing University of Chinese Medicine and the regulations of animal welfare of NIH in the USA. IBS rat models were established referring to neonatal maternal separation (NMS) pups [14] plus intracolonic infusion of 0.5% acetic acid [15]. Postnatal days 4 and 21 pups were subjected to maternal deprivation for 3 h (from 9:00 to 12:00 am), during which they were transferred from the home cage to a new plastic cage individually equipped with constant-temperature bedding (37 ± 0.5°C, heat source: a hot plate). During NMS, pups were intracolonically infused with 0.2–0.5 mL of 0.5% acetic acid daily at the same time by an angioplasty catheter (3 mm in diameter and 20 mm in length, Cordis Inc., USA) that was inserted from anus to descending colon (2 cm from anus). The control pups were left undisturbed and intracolonically infused with the same amount of saline. The pups were weaned on 22nd day. The males were selected and housed in the same cage undisturbed until they grew to 160 g (6–8 weeks old). The adult model rats in drug groups were treated with Fag (6 g/kg, 24 g/kg, resp.) and VLS#3 (VLS Pharmaceuticals. Ft, Lauderdale, FL, USA. 0.08 g/kg, 3.0 g of each tablet containing 450 billion freeze-dried bacteria) orally once daily for two weeks. The normal rats and model rats were only treated with saline.

#### 2.1.3. Evaluation of Viscera Hyperalgesia

Intense colorectal distension (CRD) is a nociceptive stimulus which enhances the contractile activities of abdomen and lowers those of limb muscles. Abdominal withdrawal reflex (AWR) evaluates the visceral sensitivity of IBS model during CRD. Rats were anaesthetized by ethyl ether, and a catheter-balloon assembly (made from latex glove; 2.5 cm in diameter and 4.0 cm in length, attached to 6 F catheter) was inserted into their descending colon (1 cm away from anus; exterior catheter was connected to a sphygmomanometer via a three-way pipe) with paraffin oil lubricant. Then the rats were put into transparent Lucite Cubicles (18 cm × 5 cm × 7 cm) and prevented from turning around. Thirty minutes after the rats' regained consciousness, air was injected into the balloon to produce the pressures of 20, 30, 40, 50, 60, 70, and 80 mmHg, respectively. Each expansion was lasted for 20 s with the intermittent of 2 min. The experiments were performed in triplicate, and the average values were recorded. Al-Chaer's method [16] was utilized as the AWR evaluation standard: 0 for no behavior responses, 1 for action pause followed by short head movement after stimulation, 2 for visible abdominal muscles without abdomen lifting off the platform, 3 for abdominal lifting off the platform, and 4 for body arching and pelvic structures or scrotum lift off.

#### 2.1.4. Total Gut PP

^51^Cr-Ethylene diamine tetraacetic acid (^51^Cr-EDTA, Perkin Elmer Life Science, Paris, France) was used as the selective paracellular permeation marker according to the Barreau's method, aiming to determine the total gut permeability towards large molecules [14]. 0.7 *μ* Ci of ^51^Cr-EDTA diluted in 500 *μ*L of saline was slowly administered orally. Then the animals were placed in metabolic cages, the faeces and urine of which were collected for 24 h. 1 mL of the collected urine and 1 mL of the 100 times diluted standard were subjected to *γ* counting measurements for 1 min to calculate the ^51^Cr-EDTA excretory rate:

(1) 51Cr−EDTA excretory rate of urine during  24 h  (%)  =((counts of urine  sample− background)×urine  volume(counts of diluted standard sample−  background)×dilution multiples)×100%.

#### 2.1.5. Evaluation of Colonic Damage/Inflammation

The myeloperoxidase (MPO) activities of colon tissues were measured as described previously [17]. Frozen pieces of distal colon (5 cm from anus) were homogenized in the phosphate buffer (in 50 mM/L, pH = 6) containing hexadecyl trimethyl ammonium bromide (0.5% w/v). Homogenates were subjected to 3 cycles of freezing and thawing (−196°C, 1 min and 37°C, 10 min) and then were further disrupted with a sonicator (Kunshan Hechuang Ultrasonic CO., LTd., China) and then centrifuged (6000 g at 4°C for 15 min). The supernatants were collected for MPO activity measurements. Protein concentrations were determined according to the modified Lowry's method (Bio Rad DC Protein Assay, France) and MPO activities were expressed as units/per gram of protein. In IFN-*γ* and TNF-*α* assay, distal colon tissues were homogenized in ice buffer solution (1mL/0.1 g) and then centrifuged for 10 min (600 g at 4°C). The supernatants were collected and detected using an ELISA kit (BIO-RAD Laboratories, Inc., USA) according to the instructions. The absorbance was measured at 492 nm.

#### 2.1.6. Colon Histological Staining

Distal colon fresh tissues were fixed in 10% paraform for 12 h, then dehydrated in 50%, 60%, 70%, 80%, 90%, and 100% alcohol separately, and embedded in paraffin at 56–58°C. The samples were cut into sections (4 *μ*M for each), deparaffinized in dimethyl benzene, gradiently dehydrated in alcohol and stained with hematoxylin, eosin (H&E), dehydrated in 70%, 90%, and 95% ethanol, and finally cleared in xylene [18]. A microscope (Nikon 80, Japan) was used to collect pictures. The positive expressing areas and the positive cells were counted by Image-J software.

#### 2.1.7. Western Blot of Colonic Claudin-l, Occludin, ZO-1 and ZO-2

Distal colonic tissues were quickly taken from sacrificed rats and stored in liquid nitrogen at −170°C and processed into a homogenate. Then lysate was added into the sample (500 *μ*L: 100 mL), to which was also added phosphatase inhibitor to protect phosphorylated proteins. The protein concentrations in the supernatants centrifuged (12500 r/min, 15 min at 4°C) and the sample were determined using a BCA protein assay kit (Pierce, Rockford, IL). The primary antibodies included rabbit polyclonal anti-ZO-1, ZO-2 (1 : 1000, Santa Cruz, Inc. America), rabbit anti-claudin-1, occludin (1 : 1000, abcam, Inc., USA) and rabbit anti-GAPDH antibody (1 : 1000, Bioworld, Inc., USA). The secondary antibody was sheep polyclonal anti-rabbit IgG (1 : 10000, IRDye800 Conjugated, Rockland, Inc., USA). LI-COR Odyssey Infrared Imaging System and LI-COR Odyssey Analysis software were utilized for scanning and analysis, respectively. 

#### 2.1.8. Immunofluorescence Staining of ZO-1 and Occludin

Rat distal colon samples were embedded in paraffin, cut into sections (thickness: 4 *μ*M), deparaffinized in dimethyl benzene, and then dehydrated in 100%, 95%, and 70% alcohol for 5 min. The antigens of the sections were restored by being placed on a metal staining rack in a pressure kettle containing boiling water to which was added phosphate buffer (0.01 mol/L, pH 6.0). The samples were processed for 5 min by gradually increasing pressure, removed, and quickly washed with cool water, followed by an additional wash 3 times with PBS (0.01 mol/L PH7.4) for 2 min. Goat serum was added to the samples, which was incubated for 20 min at 37°C, and the residual fluid was discarded. Then the samples were incubated with primary antibody (1 : 1000, rabbit anti-ZO-1, Santa Cruz, Inc. America; rabbit anti-occludin, abcam, Inc., USA) overnight at 4°C, then washed 3 times with PBS for 2 min. Thereafter, the samples were incubated with secondary antibody (1 : 200, TRITC or FITC goat anti-rabbit Ig G (H + L) conjugate, Invitrogen Inc., USA) at 37°C and then washed 3 times with PBS for 2 min. 50% glycerin was added to seal up the sections, which were then fluorescently visualized using a laser scanning confocal microscope (LSCM, Nikon Inc., Japan).

### 2.2. Caco-2 Cell Experiments

#### 2.2.1. Cell Cultures

Caco-2 cells (Cell Banks of Chinese Institute of Sciences) were grown in a culture medium consisting of Dulbecco's modified Eagle's medium (DMEM, Gibco, USA), 4.5 g/L glucose, 4.0 mmol/L glutaminate, 10^5^ U/L penicillin, 100 mg/L streptomycin, 20% Fetal Bovine Serum (FBS, Wisen, Canada) and nonessential amino-acid (Invitrogen, USA) [19]. After being digested with 0.25% trypsin and 0.02% EDTA in Ca^2+^ free and Mg^2+^ free PBS, the cells were inoculated in plug-in type 96-well plates at the density of 1 × 10^5^ cells/cm^2^ (PCF membrane, pore size 0.4 *μ*m, Millipore, USA). 

#### 2.2.2. Determination of TER

Barrier functions were evaluated by TER which reflects the colonic mucosal permeability indirectly. The apical sides of the cells were administrated with cytokines (TNF-*α* 100 ng/mL and IFN-*γ* 100 ng/mL) and incubated for 72 h. The TER of Caco-2 monolayers was measured using the Millicell-ERS (Electrical Resistance System) (Milipore, USA) after 0, 12, 24, 36, 48, and 72 h. Then the monolayers were incubated with 100 ng/mL TNF-*α*, 100 ng/mL IFN-*γ*, and Fag (0, 5, 10, 30 *μ*g/mL) for 24 h. The resistance values (RV) were measured consecutively at least 3 times. TER was determined according to:

(2) $$\begin{array}{c} \text{TER}\left(\Omega \cdot \text{c}{\text{m}}^{\text{2}}\right)=\left(R_{\text{total}}-R_{\text{blank}}\right)\times A, \end{array}$$

where *R*_total_ represents the measured RV; *R*_blank_ represents the RV without cells; *A* represents the membrane surface area [20].

#### 2.2.3. Assessment of TJs Expression by Western Blot

After digestion, the cells were incubated in 24-well plates, adhered to bottom overnight, and starved in serum-free medium for 12 h. Some cells were incubated only with Fag (0, 5, 15, 30 *μ*g/mL), while the others were incubated with TNF-*α*, IFN-*γ* and Fag (0, 5, 15, 30 *μ*g/mL) for 24 h. After intervention, NP-40 soluble protein and NP-40 insoluble protein in the cells were extracted [21] for TJs immunoblotting essay. Protease and phosphate inhibitors (Kang as Item) were added, and the protein concentrations were determined. The samples were transferred to a membrane (wet transfer) followed by SDS-Polyacrylamide gel electrophoresis, sealed with 1% bovine serum albumin at room temperature for 2 h, incubated with primary antibody (rabbit polyclonal anti-ZO-1, ZO-2, 1 : 2000, Santa Cruz, Inc., America; rabbit anti-claudin-1, occludin 1 : 2000, abcam, Inc., USA), and diluted with Tris-buffered saline (TBS) overnight at 4°C. The residual was discarded, washed 3 times with TBS containing 0.1% Tween-20 (TBST) for 5 min, administrated with secondary antibody (rabbit anti-GAPDH antibody, 1 : 1000, Bioworld, Inc., USA), diluted with TBS, and incubated at room temperature for 1 h. LI-COR Odyssey Infrared Imaging System was used for scanning.

#### 2.2.4. Fluorescein Localization of Claudin-1

Caco-2 monolayers grown on transwell filters were washed with PBS, fixed with 4.0% formaldehyde for 10 min, permeabilized in NP-40 Lysis Buffer for 30 min, and washed 3 times with PBS. The monolayers were then labeled with rabbit anti-claudin-1 antibody (1 : 2000, abcam, Inc., USA) overnight at 4°C, followed by incubation for 30 min with secondary antibodies (1 : 200, Goat Alexa Fluor 488-conjugated anti-rabbit IgG). The images were visualized using an LSCM (Nikon, Inc., Japan).

### 2.3. Statistical Analysis

All values were expressed as mean ± SEM or mean ± SD. The statistics of the two groups were compared by *t*-test or Mann-Whitney *U*-test using GraphPad-InStat, version 5.01, and *P* < 0.05 was considered as statistically significant.

## 3. Results

### 3.1. Assessment of Visceral Sensitivity to CRD

**Figure 1:**
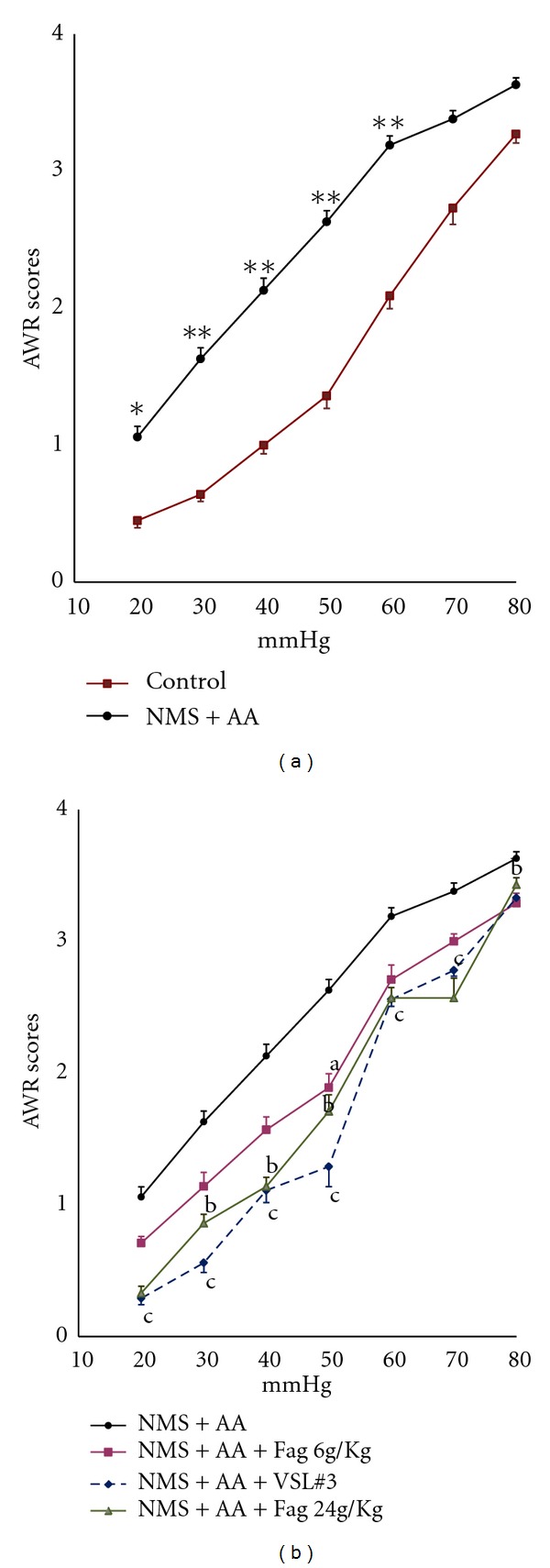
Sensitivities of the rats to CRD. (a) AWR scores of the rats in response to graded CRD. *P < 0.05, **P < 0.01, NMS + AA rats (model group) versus controls. (b) AWR scores of NMS + AA rats treated with Fag (6 g/kg, 24 g/kg) and VLS#3 ^a^ *P* < 0.05, Fag (6 g/kg) treated rats versus NMS + AA rats; ^b^ *P* < 0.05, Fag (24 g/kg) treated rats versus NMS + AA rats; ^c^ *P* < 0.05, VLS#3 treated rats versus NMS + AA rats. The data are expressed as mean ± SEM (*n* = 8–10 in each group).

The adult NMS + AA rats responded to graded CRD (20, 30, 40, 50, 60 mmHg) were significantly different from the controls (*P* < 0.05 or <0.01) (Figure 1(a)). The responses of NMS + AA and 6 g/kg Fag rats to CRD did not differ significantly except AWR decreased when CRD = 50 mmHg. The AWR score of 24 g/kg Fag rats was significantly lower (when CRD at 30, 40, 50, 80 mmHg, *P* < 0.05) than that of NMS + AA rats. Besides, the AWR score of VSL#3 rats was effectively lower (*P* < 0.05) than that of the controls (Figure 1(b)). 

### 3.2. Effect of Fag on Gut PP and Colonic Damage/Inflammation

**Figure 2:**
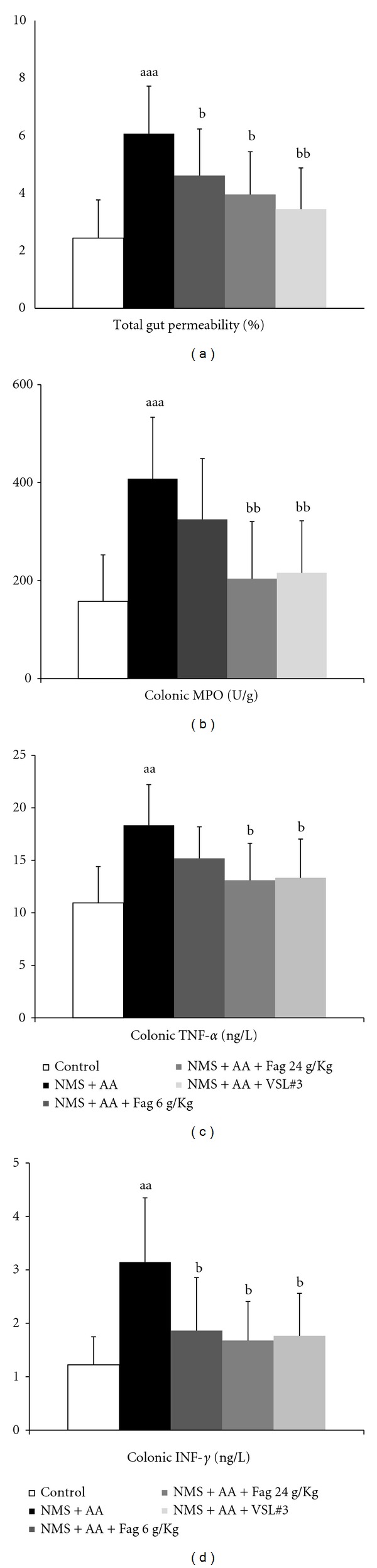
Effects of Fag on the total gut permeability (a), colonic myeloperoxidase (MPO) activity (b), colonic cytokines TNF-*α* (c) and IFN-*γ* (d) in NMS + AA rats.^aa^ *P* < 0.01, ^aaa^ *P* < 0.001, compared with control rats; ^b^ *P* < 0.05, ^bb^ *P* < 0.01, compared with NMS + AA rats. The values are expressed as mean ± SD (*n* = 8 in each group).

In NMS + AA rats, ^51^Cr-of EDTA excreted in urine over 24 h (GPP) significantly increased compared to that in control rats (*P* < 0.001), while gut PP in 6 g/kg, 24 g/kg Fag and VLS#3 rats decreased significantly (*P* < 0.05, *P* < 0.01) (Figure 2(a)). The colonic MPO activity of control rats was 157.6 ± 95 U/g of protein, which was significantly lower (*P* < 0.001) than that of NMS rats. The activities of 24 g/kg Fag and VLS#3 rats decreased (*P* < 0.01) (Figure 2(b)). The levels of TNF-*α* and IFN-*γ* in the colon of NMS + AA rats were significantly higher (*P* < 0.01) than those of the controls. TNF-*α* and IFN-*γ* in Fag treated rats exhibited a dose-dependent recovery to normal significantly compared with those in NMS rats did. VLS#3 decreased the contents of both TNF-*α* and IFN-*γ* (Figures 2(c) and 2(d)). These data demonstrated that Fag reduced gut PP by mitigating intestinal damage and inflammation.

### 3.3. Effect of Fag on Colonic Lamina Propria Inflammatory Cell Counts

**Figure 3:**
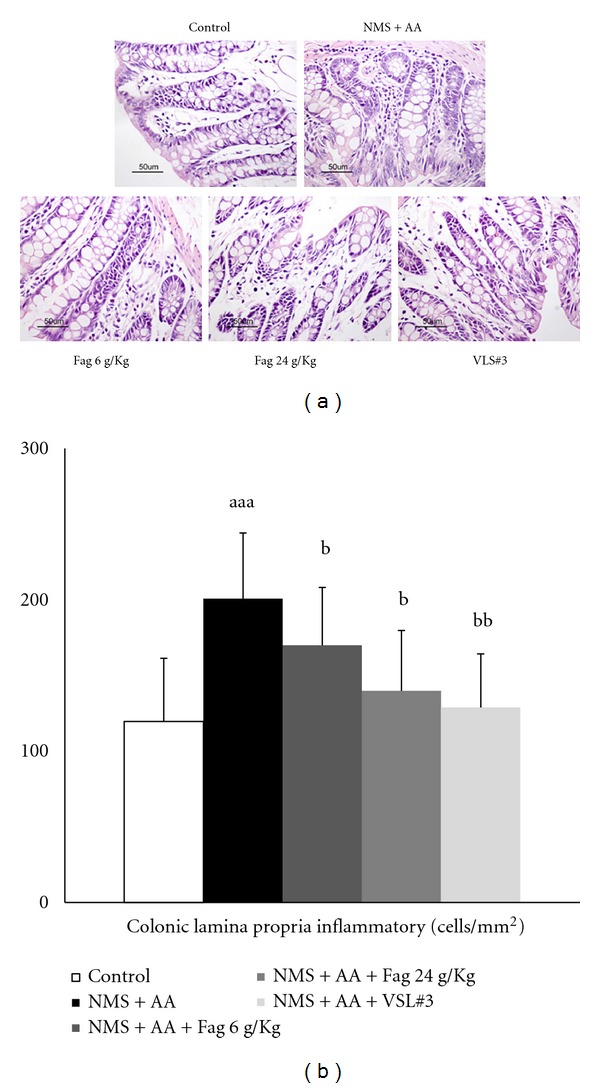
Effects of Fag treatment on colonic lamina propria inflammatory cells of rats (×200). (a) HE staining of colonic lamina propria inflammatory cells; (b) quantification of inflammatory cells. The data are expressed as cell counts per square millimeter of colon lamina propria. The values are expressed as mean ± SD (*n* = 8), ^aa^ *P* < 0.01 compared with control rats. ^b^ *P* < 0.05, ^bb^ *P* < 0.01, compared with NMS + AA rats.

HE staining shows a small number of lamina propria inflammatory cells infiltrated in the distal colon of the controls, but there were numerous inflammatory cells in NMS + AA rats (Figure 3(a)), which differed significantly (*P* < 0.01) from those in the controls. Compared with NMS + AA rats, the cell counts in Fag 24 g/kg (*P* < 0.05) and VLS#3 rats (*P* < 0.01) decreased significantly, whereas those in Fag 6 g/kg rats only decreased slightly (*P* > 0.05) (Figure 3(b)). 

### 3.4. TJs Expression of Colon Tissues

**Figure 4:**
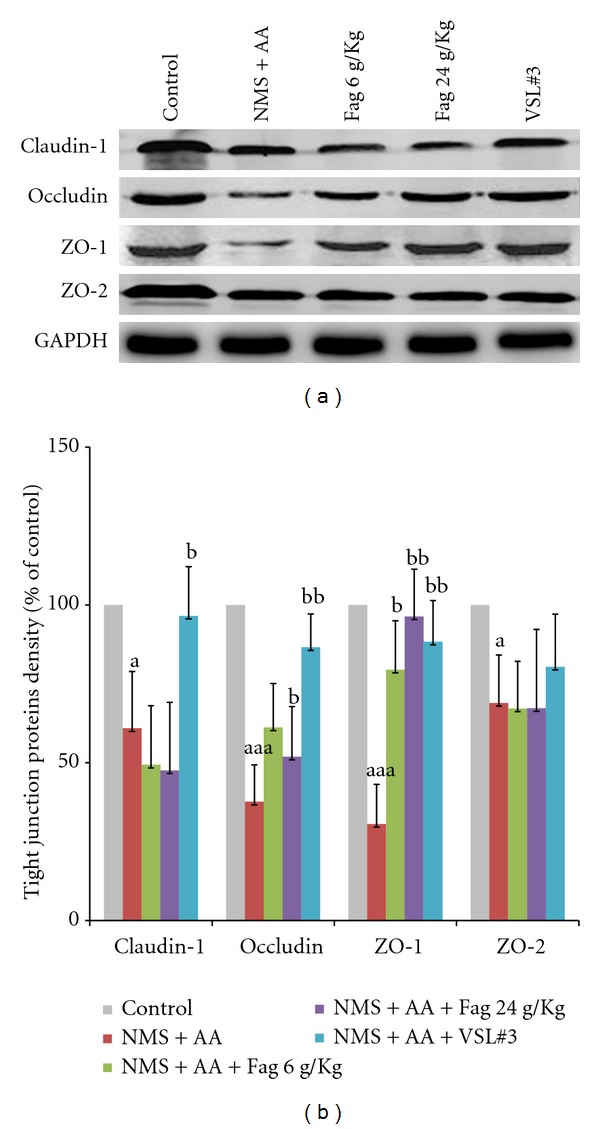
Western blot of Fag treated rat colon TJs. (a) Protein of claudin-1, occludin, ZO-1, ZO-2. (b) Relative density analysis of concentration-dependent TJs of drug-treated groups and the controls. The values are expressed as mean ± SD (*n* = 3). ^a^ *P* < 0.05, ^aaa^ *P* < 0.001, compared with control rats; ^b^ *P* < 0.05, ^bb^ *P* < 0.01 compared with NMS + AA rats.

The colonic TJs of claudin-1, occludin, ZO-1, ZO-2 were analyzed by western blotting (Figure 4(a)). Compared with the controls (100%), the total protein of the four TJs in NMS + AA rats significant decreased (*P* < 0.05). Besides, the TJs of occludin (*P* < 0.05), and ZO-1 (*P* < 0.05) in Fag-treated rats as well as the TJs of claudin-1, occludin and ZO-1 in VSL#3 rats (*P* < 0.01) were significantly higher than those in NMS + AA rats (Figure 4(b)).

**Figure 5:**
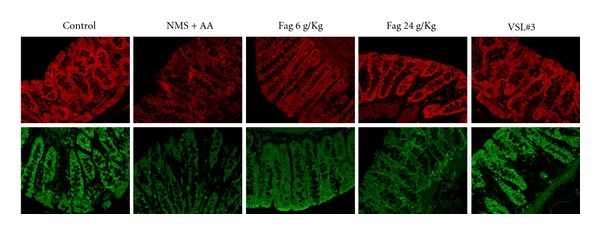
Immunofluorescence localization of ZO-1 (above) and occludin (below) in rat colon mucous membrane. The images were collected by LSCM (×40).

Moreover, we examined the expression and distribution of TJs by immunofluorescence. The four types of TJs were localized at both the surface and crypts connecting colonic cells, which is consistent with TJs distribution. However, the TJs of occludin, ZO-1 in NMS + AA rats drastically reduced. The membrane fluorescence intensities and discontinuities of NMS + AA rats declined or even, lost compared to those of the controls. In addition, 24 g/kg Fag and VLS#3 considerably prevented the loss of occludin and ZO-1, the densities of which were remarkably higher than those in NMS + AA rats (Figure 5). 

### 3.5. Effect of Fag on TER of Caco-2 Cell Monolayers

**Figure 6:**
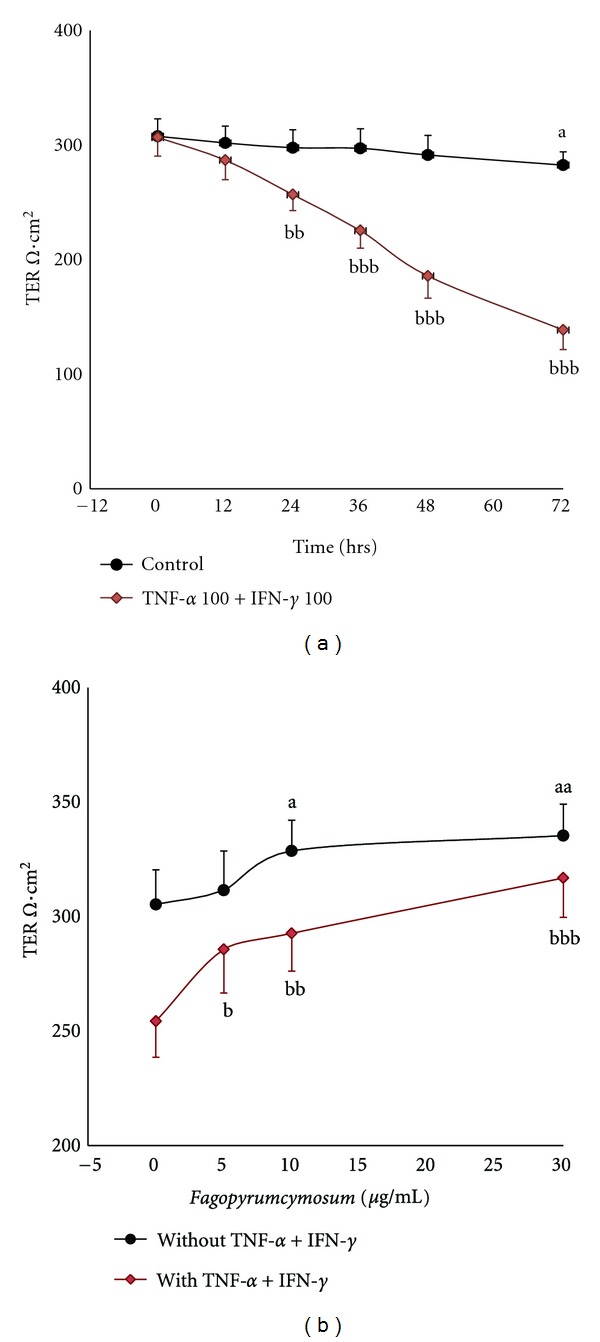
TER of Caco-2 cell monolayers (Ω*·*cm^2^). (a) Effects of treatment time of cytokines: measured at 0, 12, 24, 36, 48, and 72 h after incubating the cells with or without cytokines (100 ng/mL TNF-*α* and 100 ng/mL IFN-*γ*). All data are expressed as mean ± SD, *n* = 6. ^a^ *P* < 0.05, controls without cytokines: 72 h versus 0 h; ^bbb^ *P* < 0.001, at the same time: cells with cytokines versus controls without cytokines. (b) Effects of drug concentration gradient: measured after administrating the cells with Fag (0, 5, 10, or 30 *μ*g/mL) plus cytokines or removing cytokines during the last 24 h. The values are expressed as mean ± SD, *n* = 6. ^a^ *P* < 0.05, ^aa^ *P* < 0.01, “a” represents 10 and 30 *μ*g/mL Fag versus 0 *μ*g/mL Fag without cytokines, ^b^ *P* < 0.05, ^bb^ *P* < 0.01, ^bbb^ *P* < 0.001, “b” represents 5, 10, and 30 *μ*g/mL Fag versus 0 *μ*g/mL Fag with cytokines.

TER was lower time dependently in the cell monolayers when incubated with (TNF-*α* 100 ng/mL and IFN-*γ* 100 ng/mL at 24, 36, 48, and 72 h than controls without any intervention (*P* < 0.001) (Figure 6(a)). Without cytokines, the values were dose-dependently higher after administration of cells with Fag treatment (*P* > 0.05) for 24 h than controls (Figure 6(b)). TER was dose dependently higher (*P* < 0.05) when monolayers incubated with both cytokines (TNF-*α* 100 ng/mL and IFN-*γ* 100 ng/mL) and Fag for 24 h than controls incubated with only cytokines (Figure 6(b)).

### 3.6. Effect of Fag on TJs in Detergent-Insoluble and Soluble Fractions of Caco-2 Cells

**Figure 7:**
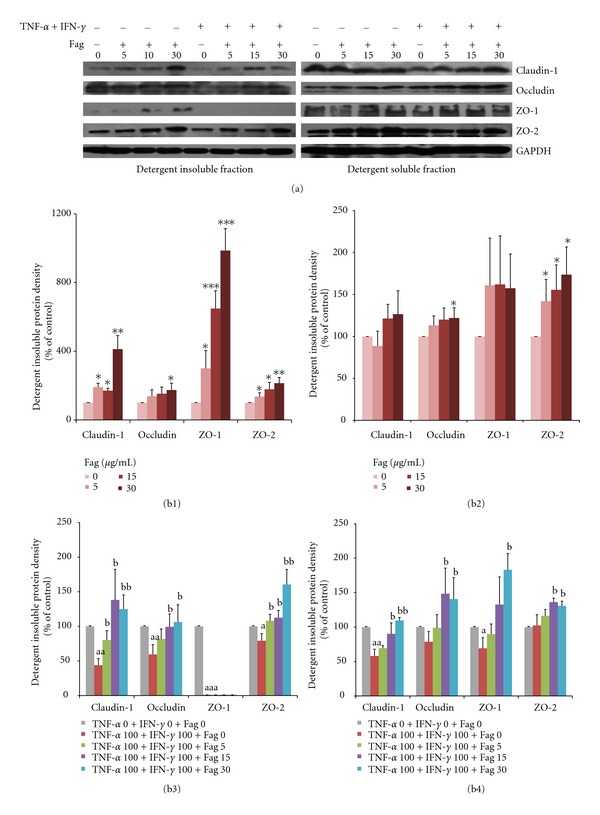
Immunoblot of TJs (claudin-1, occludin, ZO-1, ZO-2) in Caco-2 cells. (a): TJs of ZO-1, ZO-2, occludin, claudin-1 in the NP40-insoluble and soluble fractions of cells incubated with 0, 15, or 30 *μ*g/mL Fag without cytokines or with cytokines (100 ng/mL TNF-*α* and 100 ng/mL IFN-*γ*) for 24 h. (b): Relative grey values (% of the controls) of TJs. (b1)-(b3) Detergent insoluble; (b2)-(b4) detergent soluble; (b1) and (b2) were incubated with or without Fag; (b3) and (b4) were incubated with or without Fag and cytokines (100 ng/mL TNF-*α* and 100 ng/mL IFN-*γ*). The density values are normalized to those of the corresponding controls. The values are expressed as mean ± SD (*n* = 3). *P < 0.05, **P < 0.01, ***P < 0.001, the symbols indicate the differences from the controls (Fag = 0); ^a^ *P* < 0.05, ^aa^ *P* < 0.01, ^aaa^ *P* < 0.001,  ^b^ *P* < 0.05, ^bb^ *P* < 0.01, “a” represents TJs in the cells incubated with only cytokines versus controls (TNF-*α* 0 + IFN-*γ* 0 + Fag 0); “b” represents TJs in the cells incubated with Fag and cytokines versus those incubated with cytokines alone.

The protein levels of ZO-1, ZO-2, occludin, claudin-1 were observed after incubating the cells with Fag (0, 15, or 30 *μ*g/mL) in the absence of or in the presence of cytokines (100 ng/mL TNF-*α* and 100 ng/mL IFN-*γ*) for 24 h (Figure 7(a)). The densitometric analysis reveals that Fag and cytokines-free treatment increased the TJs of claudin-1, occludin, ZO-1, and ZO-2 in the NP40 detergent-insoluble fraction (Figure 7(b1)) and those of occludin and ZO-2 in the NP40-soluble fraction (Figure 7(b2)) of cells in a dose-dependent manner (*P* < 0.05). Particularly, the level of ZO-1 in the NP40-insoluble fraction was 3–9 times higher than that in the controls. When treated with cytokines, the TJs of claudin-1, occludin, ZO-1, ZO-2 in the NP40-insoluble fraction (*P* < 0.05) and those of claudin-1, ZO-1 in the NP40-soluble fraction (*P* < 0.05) were lowered. The four TJs except ZO-1 in the NP40-insoluble fraction were positively proportional to (*P* < 0.05) the doses of Fag and cytokines (Figure 7(b3)), and the TJs in the NP40-soluble fraction also increased (*P* < 0.05) when treated with cytokines and Fag (15 or 30 *μ*g/mL) (Figure 7(b4)).

### 3.7. Effect of Fag on Immunofluorescence of Claudin-1 in Caco-2 Cells

**Figure 8:**
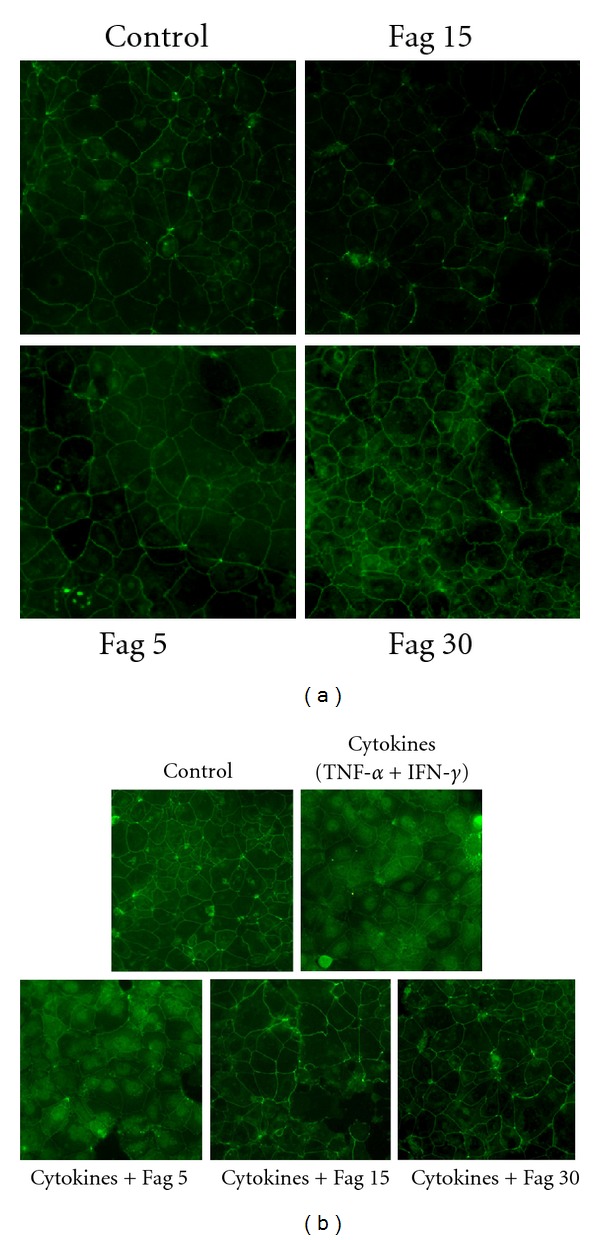
Immunofluorescence of claudin-1 in Caco-2 cell monolayers. (a) The cells were incubated with Fag (0, 5, 15, 30 *μ*g/mL) and stained by claudin-1 for 24 h. (b) The cells were incubated with Fag (0, 5, 15, 30 *μ*g/mL) and cytokines (100 ng/mL TNF-*α* and 100 ng/mL IFN-*γ*) or Fag alone for 24 h. The images were collected by LSCM (×200).

The claudin-1 in the cells incubated with Fag (15, 30 *μ*g/mL) without cytokines (100 ng/mL TNF-*α* and 100 ng/mL IFN-*γ*) for 24 h exhibited more intense immunofluorescence than the control monolayers did (Figure 8(a)). However, the fluorescence intensity decreased after being treated with cytokines. Therefore, Fag raised the fluorescence intensity of the inflammatory monolayers (Figure 8(b)).

## 4. Discussion

Previous studies have shown that NMS predisposed adult rats to colonic barrier dysfunction in response to mild stress [22] and altered the long-term colonic sensitivity to rectal distension [23]. Barreau et al. [14] found that NMS continuously altered the colonic epithelial barrier as a stress factor owing to the exaggerated expression of cytokines. Winston et al. [15] infused ten-day-old rat pups with saline containing 0.5% acetic acid intracolonically, which resulted in higher sensitivities and IFN-*γ* levels in the proximal colon in adult rats compared to those of the controls. In our study, a new IBS model of intestinal barrier dysfunction was established via repeatedly stimulating AA based on NMS, which excessively activated the intracolonic immunity. The results show that adult rats had visceral hypersensitivity and high permeability of the colonic mucosa, which are associated with the increased colonic MPO activity, lamina propria inflammatory cells, and cytokine expression, as well as the declined expressions of colonic epithelial claudin-1, occludin, ZO-1, and ZO-2.

We assume that the combination of NMS with AA activated the hypothalamic-pituitary-adrenal (HPA) axis, which may account for the increased translocation of pathogenic bacteria in gut rather than the reduced probiotic bacteria protection [7, 24]. As a result, intestinal immunity was interfered, mucosa was destructed, and PP increased ultimately [25]. 

Complicatedly structured TJs, which comprise over 50 proteins, form plasma membrane-crossing fibrils and interact with proteins in the adjoining cells and the adherens junctions that are linked to the perijunctional actomyosin ring relating to the assembly of TJs and paracellular permeability [26, 27]. TJs are controlled by various signaling pathways, including protein kinase C (PKC), mitogen-activated protein kinases (MAPK), myosin light chain kinase (MLCK), and the Rho family of small GTPases. Phosphorylated TJs are active and exhibit augmented epithelial barrier function. The phosphorylation of threonine residues in occludin plays a crucial role in the assembly and maintenance of TJs in Caco-2 and MDCK cell monolayers [28]. The assembly and integrity of adherens junctions and the level of TER depend on the phosphorylation of tyrosine residues in Caco-2 cells [29]. Nonphosphorylated occludin concentrates on the basolateral membranes while phosphorylated occludin is mainly distributed on the membranous surface as NP-40-insoluble form, almost whole cell occludin is NP-40 soluble though [30]. Fujibe et al. [31] also confirmed that phosphorylated claudin-1 was one of the main detergent-insoluble constituent in rat lung endothelial cell line RLE. TNF-*α* upregulated MLCK through initiating NF-*κ*B-mediated response, leading to ZO-1 downregulation and increased colonic epithelial permeability [32, 33]. IFN-*γ* increased the responses of epithelial monolayers to TNF-*α*, thus they synergetically disrupted TJs morphology and barrier function via MLCK up-regulation and myosin light chain (MLC) phosphorylation [34, 35]. Sappington [36] and Han [37] found that the ZO-1, ZO-3, occludin protein and ZO-1 mRNA levels decreased after exposing enterocytes to the proinflammatory mixture of TNF-*α*, IFN-*γ* and IL-1*β*.

VSL#3 treatment significantly lowers the visceral hypersensitivity and epithelial permeability of IBS rats, and inhibits the decreased expression of TJs of occludin and ZO-1 [38]. Moreover, probiotics facilitate the redistribution of TJs from the cytoplasm to the membrane [39] and TJs gene expressions [40], compete for adhesion space with intestinal pathogens [41], and antagonize cytokines-induced epithelial barrier dysfunction [42]. In this study, we found that Fag and positive control drug VLS#3 not only relieved the hyperalgesia of IBS rats, but also decreased the levels of TNF-*α* and IFN-*γ*, and promoted ZO-1, occluding or claudin-1 expression, which reduced the overall gut PP in a dose-dependent manner. In other words, Fag and VLS#3 integrated the intestinal barrier in IBS rats. 

To further investigate the Fag mechanism, we found that *in vitro* TER decreased depending on time during the 24 h of incubation of the monolayer cells with TNF-*α* and IFN-*γ*, suggesting the intestinal epithelial permeability was proportional to the duration in which cells were incubated with cytokines. Moreover, cytokines downregulated four target insoluble components of TJs, indicating inhibited phosphorylation and membrane localization. We assumed that cytokines may influence the claudin-1 and ZO-1 of monolayers at the transcriptional level due to the decreased NP-40 soluble components that represent the whole cell protein level. Nevertheless, the total levels of TJs in IBS models all decreased owing to cytokines. The immunoblotting alteration of TJs *in vitro* was not completely consistent with that *in vivo*, which may be attributed to the longer-preserved and more sophisticated colonic inflammatory microenvironment *in vivo* than those *in vitro*. The underlying mechanisms need to be further explored. 

Previous research revealed that buckwheat hulls and flour are rich in total flavonoids. Flavanols (including catechins and procyanidins) mainly contain (−)-epicatechin, (−)-epicatechin gallate, and dimeric procyanidin B-2. Flavonoids mainly contained rutin, quercetin and hyperoside [43]. The high-performance liquid chromatography (Supplemental Figure 1 avaliable on at doi:10.1155/2012/983801) reveals that Fag contains four standard procyanidins dimmers procyanidin B-2 (PB2), epicatechin, rutin and quercetin, in which procyanidin B-2 is most abundant. Both PB2 and epicatechin are classified into tannins. Procyanidins potently possess strong antioxidant activity [44] and immunomodulate IL-1*β*, IL-2, IL-4, IL-5, IFN-*γ*, and so forth [45, 46]. Green tea polyphenol (−)-epigallocatechin gallate (EGCG) prevents IFN-*γ*-induced increase of permeability in T84 and THP-1 cells [47]. However, Obara found that (−)-epicatechin gallate (ECG) induced the phosphorylation of protein phosphatase inhibitor by activating PKC*δ*, thereby inhibiting MLC phosphatase and enhancing MLC phosphorylation [48]. Thus, TJs were internalized due to actomyosin contractility [49]. Quercetin and rutin (quercetin glycosides) are pharmacologically versatile. Rutin normalizes the increased vascular permeability and fragility, prevents vascular edema [50], and lowers the risks of inflammation, lipoperoxidation, and hyperalgesia in biliary obstruction-induced acute pancreatitis [51]. Isoflavone genistein prevents TNF-*α*-induced TER reduction in colon cell line HT-29/B6, but it does not impact TER *per se* [52]. Suzuki and Hara [53] demonstrated that quercetin augmented the phosphorylation of ZO-2, claudin-1, and occludin in Caco-2 cells by inhibiting PKC*ζ* activity, which led to actomyosin redistribution, thereby refreshing the intestinal epithelial function [54].

The *in vitro* experiments show that Fag increased TER of the monolayer cells depending on concentration, and upregulated detergent-insoluble components in claudin-1, occludin, ZO-1 and ZO-2 with or without cytokines' intervention. We speculated that flavonoids such as quercetin directly intensified the phosphorylation of TJs, via integration, benefited the membrane assembly of TJs and improved intestinal barrier function. Fag might promote the expression of TJs at transcriptional level because it could sustainably restore the epithelial barrier dysfunction caused by combination treatment with TNF-*α* and IFN-*γ* and dose-dependently increase the detergent soluble components components of TJs. Probably, tannins in Fag indirectly protected the intestinal barrier from impairing by immunomodulation and prevented TJs from blocking by cytokines. The reason for the untouched total protein levels of claudin-1 and ZO-1 by Fag in the absence of cytokines is still unclear and merits further studies. So we speculated Fag may exert the direct and indirect role in reducing the colonic epithelial barrier permeability *in vivo*. Fag perhaps further avoided the exposure of intraepithelial nerve plexus and restored sensitizing process due to inflammation, which thus alleviated the visceral hypersensitivity of IBS rats. In a word, Fag may treat IBS via multi-target and multi-channel analgesia.

In summary, our study has demonstrated that Fag soothed the visceral hypersensitivity of model rats, possibly by inhibiting the colonic epithelium inflammation and injury, as well as by facilitating membrane localization and expressions of colonic epithelial TJs which strengthened the colonic barrier. This study herein provides a theoretical basis for Fag-treated IBS due to its multi-target and multichannel pharmacological values. 

## Supplementary Material

Ultra-performance liquid chromatography/mass spectrometry (UPLC/MS) chromatograms of Fagopyrum cymosum (*Trev.*) Meisn extracts (Fag). (A) Time-dependent total ion chromatogram: standards (above) and samples of Fag (below) were detected at 280 nm. (B) T SIM mass spectrogram of the four components in positive ion detection mode. 1: Procyanidin B2; Molecular Weight: 578.52024; 2: (-)-epicatechin; Molecular Weight: 290.26806; 3: Rutin; Molecular Weight: 610.5175; 4: Quercetin; Molecular Weight: 302.2357.

## References

[1] Camilleri M (2001). Management of the irritable bowel syndrome. *Gastroenterology*.

[2] Mearin F, Perelló A, Balboa A (2009). Pathogenic mechanisms of postinfectious functional gastrointestinal disorders: results 3 years after gastroenteritis. *Scandinavian Journal of Gastroenterology*.

[3] Gwee KA, Collins SM, Read NW (2003). Increased rectal mucosal expression of interleukin 1*β* in recently acquired post-infectious irritable bowel syndrome. *Gut*.

[4] Chadwick VS, Chen W, Shu D (2002). Activation of the mucosal immune system in irritable bowel syndrome. *Gastroenterology*.

[5] Cenac N, Andrews CN, Holzhausen M (2007). Role for protease activity in visceral pain in irritable bowel syndrome. *Journal of Clinical Investigation*.

[6] Gecse K, Róka R, Ferrier L (2008). Increased faecal serine protease activity in diarrhoeic IBS patients: a colonic lumenal factor impairing colonic permeability and sensitivity. *Gut*.

[7] Ferrier L, Mazelin L, Cenac N (2003). Stress-induced disruption of colonic epithelial barrier: role of interferon-*γ* and myosin light chain kinase in mice. *Gastroenterology*.

[8] Capaldo CT, Nusrat A (2009). Cytokine regulation of tight junctions. *Biochimica et Biophysica Acta*.

[9] Piche T, Barbara G, Aubert P (2009). Impaired Intestinal barrier integrity in the colon of patients with irritable bowel syndrome: involvement of soluble mediators. *Gut*.

[10] Dunlop SP, Hebden J, Campbell E (2006). Abnormal intestinal permeability in subgroups of diarrhea-predominant irritable bowel syndromes. *American Journal of Gastroenterology*.

[11] Spiller RC, Jenkins D, Thornley JP (2000). Increased rectal mucosal enteroendocrine cells, T lymphocytes, and increased gut permeability following acute *Campylobacter enteritis* and in post-dysenteric irritable bowel syndrome. *Gut*.

[12] Kong WM, Gong J, Dong L, Chen MX (2007). Changes in tight junction of intestinal mucosa in patients with irritable bowel syndrome: a study with tracing electron microscope. *Nan Fang Yi Ke Da Xue Xue Bao*.

[13] Kong WM, Gong J, Dong L, Xu JR (2007). Changes of tight junction claudin-1,-3,-4 protein expression in the intestinal mucosa in patients with irritable bowel syndrome. *Nan Fang Yi Ke Da Xue Xue Bao*.

[14] Barreau F, Ferrier L, Fioramonti J, Bueno L (2004). Neonatal maternal deprivation triggers long term alterations in colonic epithelial barrier and mucosal immunity in rats. *Gut*.

[15] Winston J, Shenoy M, Medley D, Naniwadekar A, Pasricha PJ (2007). The vanilloid receptor initiates and maintains colonic hypersensitivity induced by neonatal colon irritation in rats. *Gastroenterology*.

[16] Al-Chaer ED (2000). A new model of chronic visceral hypersensitivity in adult rats induced by colon irritation during postnatal development. *Gastroenterology*.

[17] Bradley PP, Christensen RD, Rothstein G (1982). Cellular and extracellular myeloperoxidase in pyogenic inflammation. *Blood*.

[18] Keohane J, O’Mahony C, O’Mahony L, O’Mahony S, Quigley EM, Shanahan F (2010). Irritable bowel syndrome-type symptoms in patients with inflammatory bowel disease: a real association or reflection of occult inflammation?. *The American Journal of Gastroenterology*.

[19] Ma TY, Hoa NT, Tran DD (2000). Cytochalasin B modulation of Caco-2 tight junction barrier: role of myosin light chain kinase. *American Journal of Physiology*.

[20] Ma TY, Nguyen D, Bui V, Nguyen H, Hoa N (1999). Ethanol modulation of intestinal epithelial tight junction barrier. *American Journal of Physiology*.

[21] Basuroy S, Seth A, Elias B, Naren AP, Rao R (2006). MAPK interacts with occludin and mediates EGF-induced prevention of tight junction disruption by hydrogen peroxide. *Biochemical Journal*.

[22] Söderholm JD, Yates DA, Gareau MG, Yang PC, MacQueen G, Perdue MH (2002). Neonatal maternal separation predisposes adult rats to colonic barrier dysfunction in response to mild stress. *American Journal of Physiology*.

[23] Rosztóczy A, Fioramonti J, Jármay K, Barreau F, Wittmann T, Buéno L (2003). Influence of sex and experimental protocol on the effect of maternal deprivation on rectal sensitivity to distension in the adult rat. *Neurogastroenterology and Motility*.

[24] Ando T, Brown RF, Berg RD, Dunn AJ (2000). Bacterial translocation can increase plasma corticosterone and brain catecholamine and indoleamine metabolism. *American Journal of Physiology*.

[25] Quigley EMM (2005). Disturbances of motility and visceral hypersensitivity in irritable bowel syndrome: biological markers or epiphenomenon. *Gastroenterology Clinics of North America*.

[26] Furuse M (2010). Molecular basis of the core structure of tight junctions. *Cold Spring Harbor Perspectives in Biology*.

[27] Ulluwishewa D, Anderson RC, McNabb WC, Moughan PJ, Wells JM, Roy NC (2011). Regulation of tight junction permeability by intestinal bacteria and dietary components. *Journal of Nutrition*.

[28] Suzuki T, Elias BC, Seth A (2009). PKC*η* regulates occludin phosphorylation and epithelial tight junction integrity. *Proceedings of the National Academy of Sciences of the United States of America*.

[29] Morgado-Díaz JA, De Souza W (2001). Evidence that increased tyrosine phosphorylation causes disassembly of adherens junctions but does not perturb paracellular permeability in Caco-2 cells. *Tissue and Cell*.

[30] Sakakibara A, Furuse M, Saitou M, Ando-Akatsuka Y, Tsukita S (1997). Possible involvement of phosphorylation of occludin in tight junction formation. *Journal of Cell Biology*.

[31] Fujibe M, Chiba H, Kojima T (2004). Thr203 of claudin-1, a putative phosphorylation site for MAP kinase, is required to promote the barrier function of tight junctions. *Experimental Cell Research*.

[32] Zareie M, McKay DM, Kovarik GG, Perdue MH (1998). Monocyte/macrophages evoke epithelial dysfunction: indirect role of tumor necrosis factor-*α*. *American Journal of Physiology*.

[33] Mashukova A, Wald FA, Salas PJ (2011). Tumor necrosis factor alpha and inflammation disrupt the polarity complex in intestinal epithelial cells by a posttranslational mechanism. *Molecular and Cellular Biology*.

[34] Graham WV, Wang F, Clayburgh DR (2006). Tumor necrosis factor-induced long myosin light chain kinase transcription is regulated by differentiation-dependent signaling events: characterization of the human long myosin light chain kinase promoter. *Journal of Biological Chemistry*.

[35] Wang F, Graham WV, Wang Y, Witkowski ED, Schwarz BT, Turner JR (2005). Interferon-*γ* and tumor necrosis factor-*α* synergize to induce intestinal epithelial barrier dysfunction by up-regulating myosin light chain kinase expression. *American Journal of Pathology*.

[36] Sappington PL, Han X, Yang R, Delude RL, Fink MP (2003). Ethyl pyruvate ameliorates intestinal epithelial barrier dysfunction in endotoxemic mice and immunostimulated caco-2 enterocytic monolayers. *Journal of Pharmacology and Experimental Therapeutics*.

[37] Han X, Fink MP, Delude RL (2003). Proinflammatory cytokines cause NO*-dependent and -independent changes in expression and localization of tight junction proteins in intestinal epithelial cells. *Shock*.

[38] Dai C, Guandalini S, Zhao D-H, Jiang M (2012). Antinociceptive effect of VSL#3 on visceral hypersensitivity in a rat model of irritable bowel syndrome: a possible action through nitric oxide pathway and enhance barrier function. *Molecular and Cellular Biochemistry*.

[39] Zyrek AA, Cichon C, Helms S, Enders C, Sonnenborn U, Schmidt MA (2007). Molecular mechanisms underlying the probiotic effects of *Escherichia coli* Nissle 1917 involve ZO-2 and PKC*ζ* redistribution resulting in tight junction and epithelial barrier repair. *Cellular Microbiology*.

[40] Anderson RC, Cookson AL, McNabb WC (2010). Lactobacillus plantarum MB452 enhances the function of the intestinal barrier by increasing the expression levels of genes involved in tight junction formation. *BMC Microbiology*.

[41] Putaala H, Salusjärvi T, Nordström M (2008). Effect of four probiotic strains and *Escherichia coli* O157:H7 on tight junction integrity and cyclo-oxygenase expression. *Research in Microbiology*.

[42] Resta-Lenert S, Barrett KE (2006). Probiotics and commensals reverse TNF-*α*- and IFN-*γ*-induced dysfunction in human intestinal epithelial cells. *Gastroenterology*.

[43] Quettier-Deleu C, Gressier B, Vasseur J (2000). Phenolic compounds and antioxidant activities of buckwheat (*Fagopyrum esculentum* Moench) hulls and flour. *Journal of Ethnopharmacology*.

[44] Da Silva Porto PAL, Laranjinha JAN, De Freitas VAP (2003). Antioxidant protection of low density lipoprotein by procyanidins: Structure/activity relationships. *Biochemical Pharmacology*.

[45] Mao TK, Van De Water J, Keen CL, Schmitz HH, Gershwin ME (2002). Effect of cocoa flavanols and their related oligomers on the secretion of interleukin-5 in peripheral blood mononuclear cells. *Journal of Medicinal Food*.

[46] Takano F, Takata T, Yoshihara A, Nakamura Y, Arima Y, Ohta T (2007). Aqueous extract of peanut skin and its main constituent procyanidin A1 suppress serum IgE and IgG1 levels in mice-immunized with ovalbumin. *Biological and Pharmaceutical Bulletin*.

[47] Watson JL, Ansari S, Cameron H, Wang A, Akhtar M, McKay DM (2004). Green tea polyphenol (−)-epigallocatechin gallate blocks epithelial barrier dysfunction provoked by IFN-*γ* but not by IL-4. *American Journal of Physiology*.

[48] Obara K, Ukai K, Ishikawa T (2011). Mechanism of potentiation by tea epigallocatechin of contraction in porcine coronary artery: the role of protein kinase Cdelta-mediated CPI-17 phosphorylation. *European Journal of Pharmacology*.

[49] Kaneko-Kawano T, Takasu F, Naoki H (2012). Dynamic regulation of myosin light chain phosphorylation by Rho-kinase. *PLoS One*.

[50] Ihme N, Kiesewetter H, Jung F (1996). Leg oedema protection from a buckwheat herb tea in patients with chronic venous insufficiency: a single-centre, randomised, double-blind, placebo controlled clinical trial. *European Journal of Clinical Pharmacology*.

[51] Santana DG, Santos CA, Santos ADC (2012). Beneficial effects of the ethanol extract of *Caesalpinia pyramidalis* on the inflammatory response and abdominal hyperalgesia in rats with acute pancreatitis. *Journal of Ethnopharmacology*.

[52] Schmitz H, Fromm M, Bentzel CJ (1999). Tumor necrosis factor-alpha (TNF*α*) regulates the epithelial barrier in the human intestinal cell line HT-29/B6. *Journal of Cell Science*.

[53] Suzuki T, Hara H (2009). Quercetin enhances intestinal barrier function through the assembly of zonnula occludens-2, occludin, and claudin-1 and the expression of claudin-4 in caco-2 cells. *Journal of Nutrition*.

[54] Amasheh M, Schlichter S, Amasheh S (2008). Quercetin enhances epithelial barrier function and increases claudin-4 expression in Caco-2 cells. *Journal of Nutrition*.

