# Supplementary material for: In Vivo and In Vitro Antinociceptive Effect of Fagopyrum cymosum (Trev.) Meisn Extracts: A Possible Action by Recovering Intestinal Barrier Dysfunction
Source: Evid Based Complement Alternat Med. 2012 Dec 17;2012:983801. doi: 10.1155/2012/983801 (PMC3541707; doi:10.1155/2012/983801)
Supplement: Supplementary file 1 — Ultra-performance liquid chromatography/mass spectrometry (UPLC/MS) chromatograms of Fagopyrum cymosum (Trev.) Meisn extracts (Fag). (A) Time-dependent total ion chromatogram: standards (above) and samples of Fag (below) were detected at 280 nm. (B) T SIM mass spectrogram of the four components in positive ion detection mode. 1: Procyanidin B2; Molecular Weight: 578.52024; 2: (-)-epicatechin; Molecular Weight: 290.26806; 3: Rutin; Molecular Weight: 610.5175; 4: Quercetin; Molecular Weight: 302.2357. [file 983801.f1.docx]

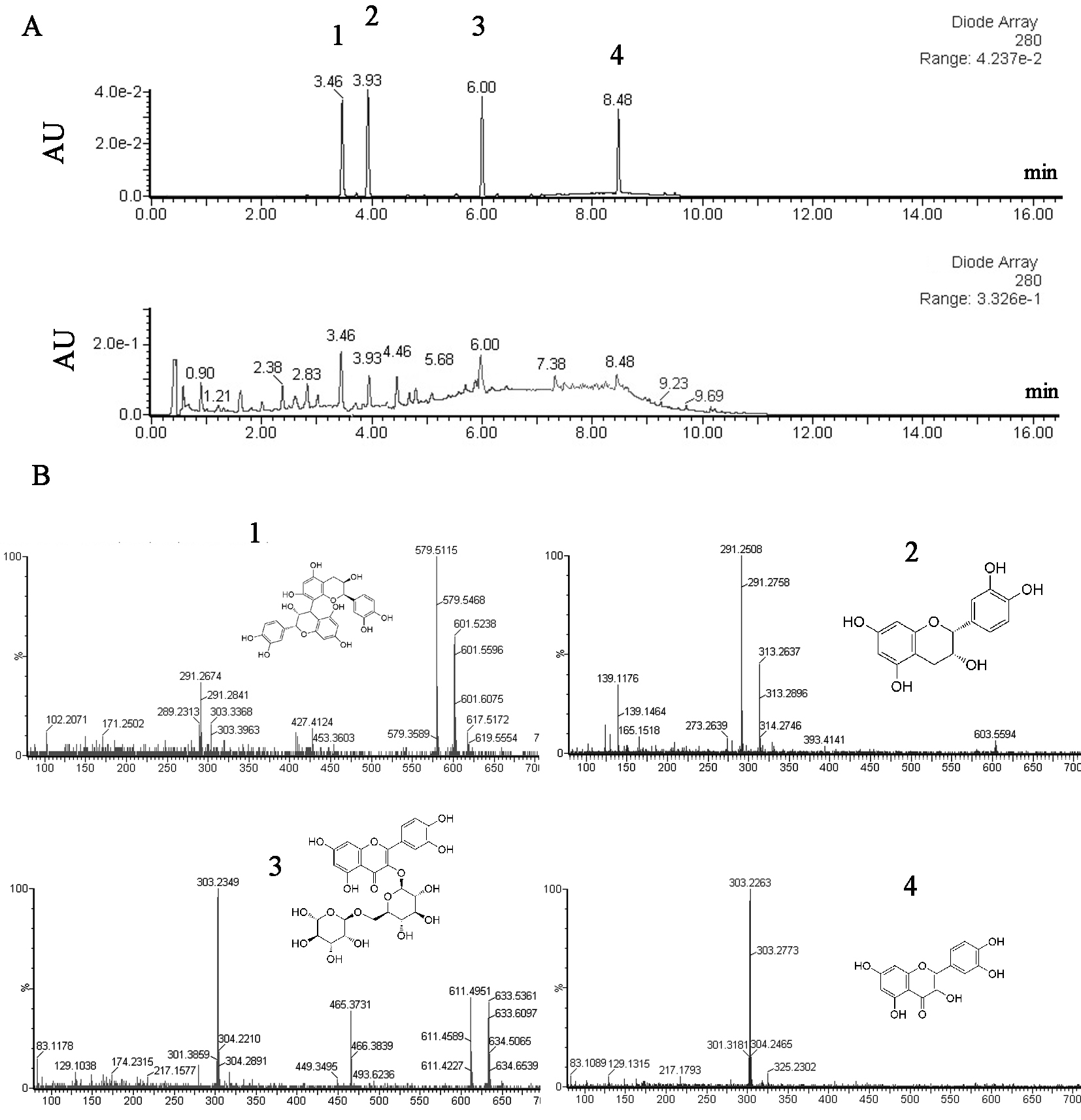


**Supplementary Fig**: Ultra-performance liquid chromatography/mass spectrometry (UPLC/MS) chromatograms of *Fagopyrum cymosum (Trev.) Meisn* extracts (Fag). (A) Time-dependent total ion chromatogram: standards (above) and samples of Fag (below) were detected at 280 nm. (B) T SIM mass-spectrogram of the four components in positive ion detection mode. 1: Procyanidin B2; Molecular Weight: 578.52024; 2: (-)-epicatechin; Molecular Weight: 290.26806; 3: Rutin; Molecular Weight: 610.5175; 4: Quercetin; Molecular Weight: 302.2357.
